# Research on Coal and Gangue Recognition Based on the Improved YOLOv7-Tiny Target Detection Algorithm

**DOI:** 10.3390/s24020456

**Published:** 2024-01-11

**Authors:** Yiping Sui, Lei Zhang, Zhipeng Sun, Weixun Yi, Meng Wang

**Affiliations:** 1 College of Coal Engineering, Shanxi Datong University, Datong 037003, China; 210857002126@sxdtdx.edu.cn (Y.S.); 220857002150@sxdtdx.edu.cn (Z.S.); 230857002147@sxdtdx.edu.cn (W.Y.); 230857002119@sxdtdx.edu.cn (M.W.); 2 Key Laboratory of Deep Coal Mining of the Ministry of Education, School of Mines, China University of Mining and Technology, Xuzhou 221116, China

**Keywords:** artificial intelligence algorithm, recognition of coal and gangue, YOLOv7-tiny model, model performance evaluation index, ablation experiment

## Abstract

The recognition technology of coal and gangue is one of the key technologies of intelligent mine construction. Aiming at the problems of the low accuracy of coal and gangue recognition models and the difficult recognition of small-target coal and gangue caused by low-illumination and high-dust environments in the coal mine working face, a coal and gangue recognition model based on the improved YOLOv7-tiny target detection algorithm is proposed. This paper proposes three model improvement methods. The coordinate attention mechanism is introduced to improve the feature expression ability of the model. The contextual transformer module is added after the spatial pyramid pooling structure to improve the feature extraction ability of the model. Based on the idea of the weighted bidirectional feature pyramid, the four branch modules in the high-efficiency layer aggregation network are weighted and cascaded to improve the recognition ability of the model for useful features. The experimental results show that the average precision mean of the improved YOLOv7-tiny model is 97.54%, and the FPS is 24.73 f·s^−1^. Compared with the Faster-RCNN, YOLOv3, YOLOv4, YOLOv4-VGG, YOLOv5s, YOLOv7, and YOLOv7-tiny models, the improved YOLOv7-tiny model has the highest recognition rate and the fastest recognition speed. Finally, the improved YOLOv7-tiny model is verified by field tests in coal mines, which provides an effective technical means for the accurate identification of coal and gangue.

## 1. Introduction

The intelligent construction of the mine provides strong support for safe and efficient mining in coal mines, and the intelligent mining of the fully mechanized mining face greatly improves coal production [1,2]. Due to the complexity of the geological conditions of coal mining in China, raw coal is mixed with a 20−25% proportion of gangue, which affects the quality of the coal and increases the cost of subsequent coal washing and screening [3,4]. At the same time, the mixed combustion of coal and gangue will reduce the calorific value of the coal and emit harmful gases that pollute the environment. Traditional coal–gangue separation mainly relies on manual separation and equipment separation. Manual separation is not only time-consuming and laborious, but also has great uncertainty in the separation results [5]. With equipment separation, it is easy to cause environmental pollution. Therefore, coal and gangue identification will become the key support for the intelligent construction of coal mines.

Many experts and scholars have performed a lot of research on the identification of coal and gangue. The commonly used methods mainly include infrared thermal imaging recognition, electromagnetic wave recognition, reflection spectrum recognition, ultrasonic recognition, vibration signal recognition, and so on. Infrared thermal imaging recognition technology has a high recognition accuracy for coal and gangue, but it is easy to produce safety hazards. Electromagnetic wave recognition technology is used to identify coal and gangue according to differences in parameters such as the echo time difference and the frequency of electromagnetic waves. It has a high recognition rate, but the cost is relatively high. Reflection spectrum identification technology uses the differences in the acoustic transmission, acoustic reflection, and echo signal intensity produced by ultrasonic wave propagation in coal and gangue to identify them. This technology is vulnerable to the adverse effects of dust in coal mines. Vibration signal recognition technology is used to identify coal and gangue by detecting the vibration response of mining machinery during mining. However, due to the randomness of vibration signals, it will cause difficulties in practical applications.

With the rapid development of artificial intelligence technology, the target detection algorithm in the field of computational vision has provided technical support for the accurate and rapid identification of coal–gangue [6]. Image-based coal and gangue recognition belongs to noncontact recognition [7,8]. After the shearer cuts the coal wall, the falling coal is transported through the scraper conveyor or belt conveyor [9]. By obtaining the coal and gangue images on the conveyor in real time, the coal and gangue can be tracked and identified. The working face of the coal mine has the environmental characteristics of low illumination and high dust. The target detection algorithm has a low accuracy for the recognition of unclear coal–gangue images, and lacks the effective recognition of small-target coal–gangue [10,11]. The YOLOv7-tiny is a typical model of YOLO series target detection algorithms. It has a high detection speed and recognition accuracy, and can effectively identify small-target objects. In order to solve the problem of the accurate recognition of coal and gangue in complex environments, this paper proposes a coal and gangue recognition model based on the YOLOv7-tiny target detection algorithm. In order to improve the recognition rate and recognition speed of the YOLOv7-tiny model for coal and gangue, this paper improves the model from three aspects. The coordinate attention mechanism is added to the backbone feature extraction network of the model to improve the positioning ability of the model to coal and gangue on the conveyor belt. The context converter module is inserted into the spatial pyramid cross-stage partial channel module to improve the model’s ability to extract image features, thereby improving the model’s recognition rate. Based on the idea of the weighted bidirectional feature pyramid, the four branch modules in the efficient aggregation network module are weighted and cascaded to achieve the purpose of reducing the model size and improving the model recognition speed. After completing these three improvements, the improved YOLOv7-tiny model was finally obtained to identify coal and gangue.

Image-based coal and gangue recognition technology has the characteristics of low cost and strong real-time performance. However, the low-illumination and high-dust environment in the coal mine will lead to the low recognition accuracy of most target detection algorithm models for coal and gangue, or difficulty in identifying small-target coal or gangue. The purpose of this paper is to design a coal–gangue recognition model based on the YOLOv7-tiny target detection algorithm to complete the recognition of coal and gangue on the belt conveyor.

With respect to innovation, the research content of this paper provides, for the first time, the use of a lightweight version of the YOLOv7-tiny model to solve the problem of coal and gangue identification. Because the characteristics of the lightweight model include a small amount of calculation, the faster detection speed of the target and the identification of coal and gangue on a high-speed belt conveyor can be realized. Then, many aspects of the lightweight model are improved, so to improve the recognition rate of coal and gangue while ensuring that the model has a faster recognition speed, and to realize the accurate and efficient recognition of coal and gangue on the belt conveyor in the complex environment of the coal mine.

## 2. Improvement of the YOLOv7-Tiny Coal and Gangue Recognition Model

The YOLOv7 is a typical representative of target detection algorithms. It has the characteristics of a fast recognition speed and a high precision [12]. It is widely used in real-time target detection scenarios. The environmental characteristics of low illumination and high dust in the underground working face of the coal mine easily cause the problems of a high similarity and a low recognition of coal and gangue characteristics [13,14]. The gradient information in the ELAN module and the MP module of the YOLOv7 model backbone network and neck network is reused in large quantities, which causes a large computational overhead to the model, resulting in a reduction in its inference speed, which cannot meet the needs of fast and real-time coal and gangue recognition in the underground working face of coal mines. At the same time, the feature fusion network of the YOLOv7 algorithm adopts the ordinary convolution module, which lacks the deep extraction of feature maps of different scales and has a poor recognition effect on small-target coal and gangue with a complex image background [15]. In order to solve the above problems, this paper proposes a coal and gangue recognition model based on the improved YOLOv7-tiny.

The YOLOv7-tiny is a lightweight model which is simplified from the YOLOv7 model. It compresses the model on the basis of ensuring the detection accuracy, so that it has fewer parameters and a faster detection speed, which is suitable for the needs of rapid detection in this paper. At the same time, the smaller model is also suitable for deployment on the detection pipeline, so this paper chooses to use the YOLOv7-tiny as the basic network for improvement. The YOLOv7-tiny mainly includes a feature extraction network (backbone), a feature fusion network (neck), and a feature detection output layer (head) [16,17]. At the input end, mosaic technology is used to randomly splice the input images, enrich the image background, and improve the robustness of the network. The backbone is mainly composed of efficient layer aggregation networks. By controlling the shortest and longest gradient path, the deeper modules can learn more effectively and converge better [18,19]. The model uses the path aggregation network (PANet) structure in the neck part to fuse the shallow features with the deep features to enhance these features, and uses standard convolution to adjust the number of channels in the head part to obtain the final detection results.

**Figure 1:**
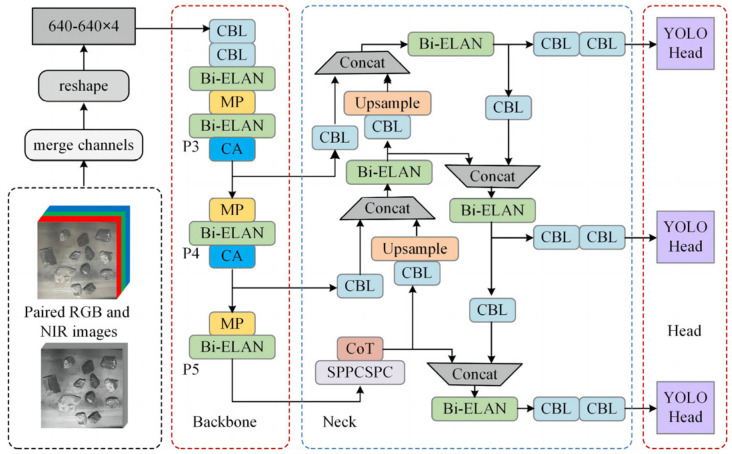
Improved YOLOv7-tiny network structure.

The improved YOLOv7-tiny model structure is shown in Figure 1. Paired RGB images and NIR images are merged through channels to obtain four-channel images. After the image size reset, 640 × 640 × 4 images are obtained for the input network. The backbone is used to extract features from the four-channel images and generate three feature maps, P3, P4, and P5, with different scales. The obtained feature map is input into the PANet, and multiscale feature fusion is performed along the top-down and bottom-up paths. The high-level semantic information and low-level positioning information are transmitted in two directions to enhance the representation ability of the backbone network. Finally, the fused features are input into the YOLO head for the regression of the prediction box and the judgment of the target category. The main modules contained in the model are as follows: CBL is the abbreviation for convolutions with batch normalization and a leaky ReLU. It is a basic module commonly used in convolutional neural networks. Its advantage is that it can effectively reduce overfitting, and can also improve the generalization ability and accelerate the training process of the model. ELAN is the abbreviation for the high-efficiency layer aggregation network. Its role is to solve the problem of the gradual deterioration of the convergence of the deep model when the model is scaled. The Bi-ELAN module is a new module that uses the idea of the bidirectional feature pyramid network to improve the ELAN module. The MP module has two branches, which are the maximum pooling and the change of the number of channels. Its function is to perform downsampling. The results of the two branches are added together to obtain the super downsampling result.

**Figure 2:**
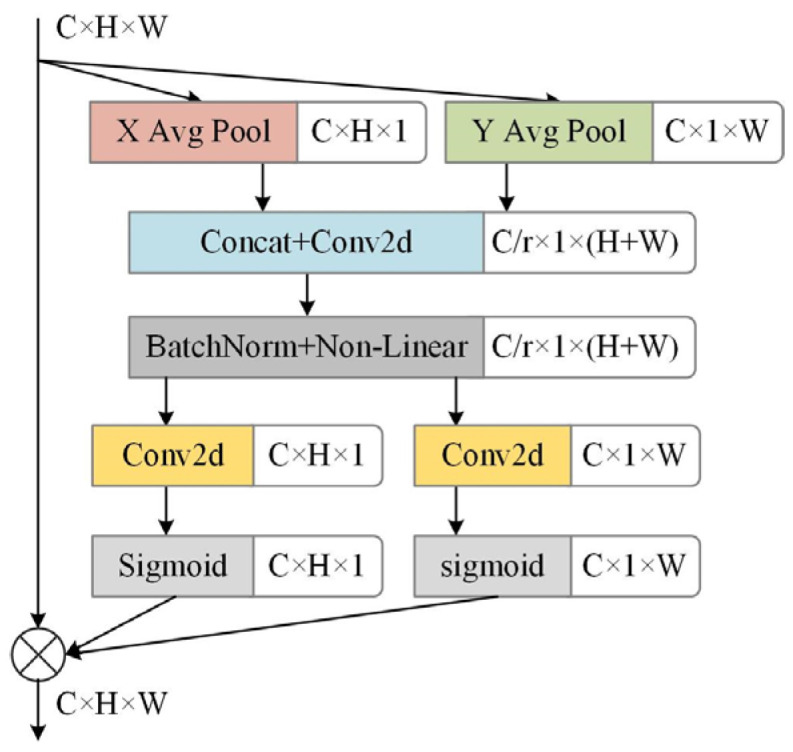
Coordinate attention mechanism module structure diagram.

In order to improve the recognition ability of the model to coal and gangue, the coordinate attention mechanism is added after the P3 and P4 feature maps of the backbone feature extraction network, and its structure is shown in Figure 2. In Figure 2, C, H, and W represent the number of channels, and the height and width of the input image, respectively; Conv2d is a convolution function and Sigmoid is an activation function; r denotes the reduction ratio of the channel attention block size. P3 and P4 are two feature maps of different scales extracted by the model. The coordinate attention mechanism first uses the average pooling in the horizontal and vertical directions to encode the feature map, and then cascades its channels [20]. Average pooling divides the input image into several rectangular regions and outputs the average value of all elements for each subregion. Cascade channels use multiple convolution layers to extract more highly abstract features or obtain more accurate regression results. After the convolution and activation operations, it is divided into two tensors; then, the convolution and activation operations are performed, respectively, to obtain two coordinate attention maps in the width direction and the horizontal direction. These two coordinate attention maps are a pair of direction-aware and position-sensitive feature maps encoded by the feature maps extracted by the model. The final output is the multiplication of the original branch and these two attention maps. The attention mechanism of the coordinate attention mechanism considers both the position relationship and the channel relationship, captures the cross-channel information, and also contains direction and position information so that the model can more accurately locate the position of the coal and gangue on the conveyor belt, and then help the model to better complete the identification of the coal and gangue.

In order to improve the detection ability of the model to the characteristics of the coal and gangue, it is necessary to further obtain the characteristics from the global receptive field. After inserting the contextual transformer into the spatial pyramid cross-stage partial channel module, based on the global interaction mechanism of the transformer, the effective receptive field can be rapidly expanded and the global dependence between the input data can be modeled through the self-attention mechanism, thereby obtaining more feature information [21]. For the feature matrix *X* with an input size of *H* × *W* × *C*, the feature mapping operation is performed to obtain *K*, *Q*, and *V*, as shown below:

(1) $$K=X,Q=X,V=XW_{V}$$

In the formula, *W_V_* is the feature mapping matrix that transforms *X* into *V*. Then, *K* is convoluted with a convolution kernel of size 3 to obtain the feature map *K*_1_, reflecting the static context information of the local adjacent position. The attention matrix *A* is obtained by using *K*_1_ and *Q*, and the calculation formula is as follows:

(2) $$A=\left[K_{1},Q\right]W_{\theta }W_{\delta }$$

In the formula, [*K*_1_,*Q*] represents the cascading operation, and *W_θ_* and *W_δ_* are two 1 × 1 convolution operations. Based on the obtained attention matrix *A* and *V* obtained by the feature mapping, the feature mapping *K*_2_ for capturing the dynamic context feature interaction is calculated. The calculation formula is as follows:

(3) $$K_{2}=V⊗A$$

**Figure 3:**
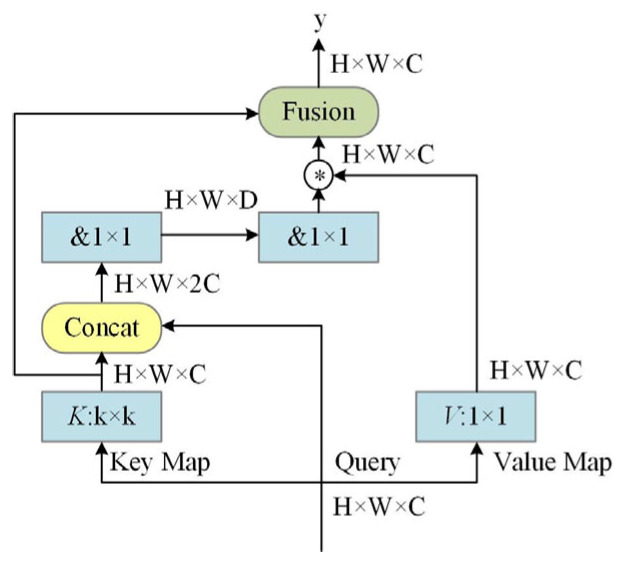
Contextual transformer module structure diagram.

In the formula, ⨂ represents the local matrix multiplication operation. The final output *Y* is the fusion of the static context representation *K*_1_ and the dynamic context representation *K*_2_. The contextual transformer module is a self-attention learning module that can fully exploit the adjacent context information and global information, and its structure is shown in Figure 3. In Figure 3, & denotes the 1 × 1 convolution operation with activation function, and ⊛ denotes the 1 × 1 convolution operation without activation function. In this paper, the module is added after the spatial pyramid pooling structure to extract global features while ensuring a small increase in the number of parameters. At the same time, certain context information is obtained, so that the feature extraction ability is enhanced.

**Figure 4:**
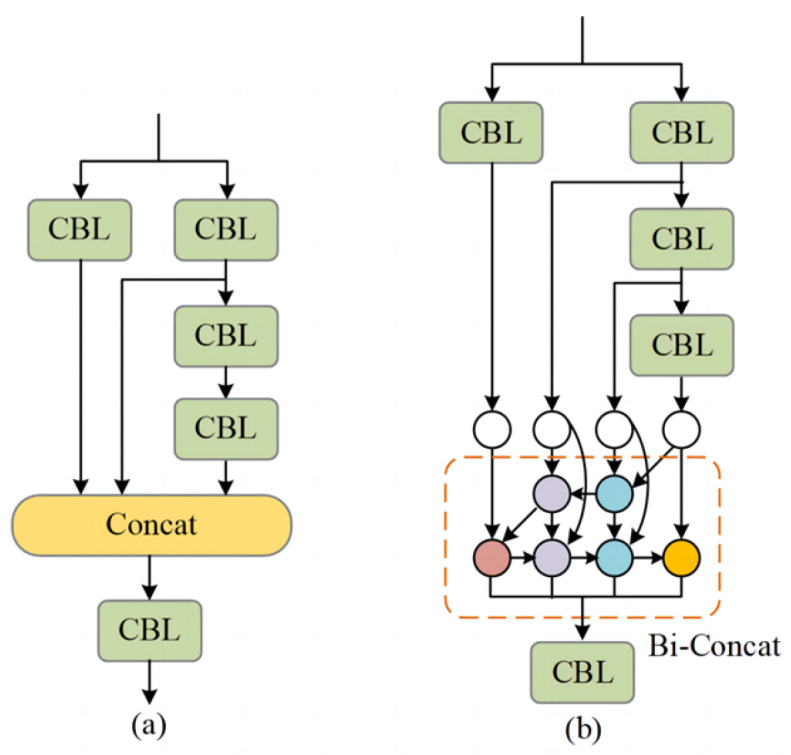
ELAN and Bi-ELAN structures. (**a**) ELAN; (**b**) Bi-ELAN.

The YOLOv7-tiny simplifies the extended efficient layer aggregation networks (E-ELANs) in YOLOv7 to an efficient aggregation network ELAN in order to reduce the model size and improve the reasoning speed. Although this structure can improve the fusion efficiency while ensuring the shortest gradient path, it is difficult to fully fuse the feature information by only splicing the channels between the upper and lower layers of the network. Therefore, based on the idea of the bidirectional feature pyramid network (Bi-FPN), the four branch modules in the ELAN module are weighted and cascaded to automatically allocate weights so as to improve the feature extraction ability of the module and the recognition ability of useful features. The new module is named the Bi-ELAN. The structure of the original ELAN and Bi-ELAN is shown in Figure 4. The Bi-ELAN replaces the Concat cascade operation in the original ELAN with the Bi-Concat weighted cascade.

## 3. Experimental Analysis

The deep-learning framework Pytorch version used in the construction and improvement of the algorithm in this paper is 1.7.1, the CUDA version is 11.0, the operating system version is Ubuntu 20.04.4 LTS, and the GPU is the NVIDA GeForce RTX 3070.

### 3.1. Model Performance Evaluation Index

In this paper, the coal and gangue recognition model will be comprehensively evaluated from the aspects of the recognition accuracy and speed. The precision, recall, *F*1 value, average precision (AP), and mean average precision (mAP) are used as the evaluation indices of the recognition accuracy before and after the improvement of the YOLOv7-tiny model. Some of the evaluation index formulae are as follows:

(4) $$P_{r}=\frac{T_{P}}{T_{P}+F_{P}}\times 100\%$$

(5) $$R_{c}=\frac{T_{p}}{T_{p}+F_{N}}\times 100\%$$

(6) $$F1=\frac{2*P_{r}*R_{c}}{P_{r}+R_{c}}\times 100\%$$

(7) $$AP=\int_{0}^{1}P_{r}\left(R_{c}\right)dR_{c}$$

(8) $$mAP=\frac{\sum_{1}^{N}AP}{N}$$

In the above formulae, *TP* represents that the positive sample recognition result is the number of positive samples; *FP* represents that the negative sample recognition result is the number of positive samples; *FN* represents that the positive sample recognition result is the number of negative samples; *N* is the number of categories in the dataset, where *N* = 2 is coal and gangue; *AP* is the area enclosed by the PR–RC curve. The precision rate *P_r_*, recall rate *R_c_*, *F*1, and *AP* can reflect the recognition accuracy of the model for a single category (coal or gangue) [22,23]. The average precision mean *mAP* is the arithmetic mean of the *AP* value of coal and gangue, which can reflect the overall accuracy of the model for all categories (coal and gangue) [24].

### 3.2. Model Training and Recognition Results

In this paper, three datasets are needed to study the identification of coal and gangue, which include a public dataset, a laboratory self-made coal and gangue dataset, and a coal and gangue dataset of the underground working face of a coal mine. The public dataset contains a total of 7261 labeled images in 10 categories. The self-made coal and gangue dataset in the laboratory was sampled from the 8304 working face of the Tashan Coal Mine in Shanxi Province. More than 800 images were collected by an explosion-proof camera, according to the ratio of coal and gangue of 1:1, with different placement positions, camera distances, light intensities, and combinations, and expanded to 4872 images by a data enhancement method. The coal and gangue dataset of the underground working face of the coal mine was sampled at the 5013 fully mechanized mining face of the Xiaoyu Coal Mine in Shanxi Province. The coal and gangue were dynamically sampled on the belt conveyor through the explosion-proof camera. A total of more than 1000 images were collected, and expanded to 6397 images through the data enhancement method.

**Figure 5:**
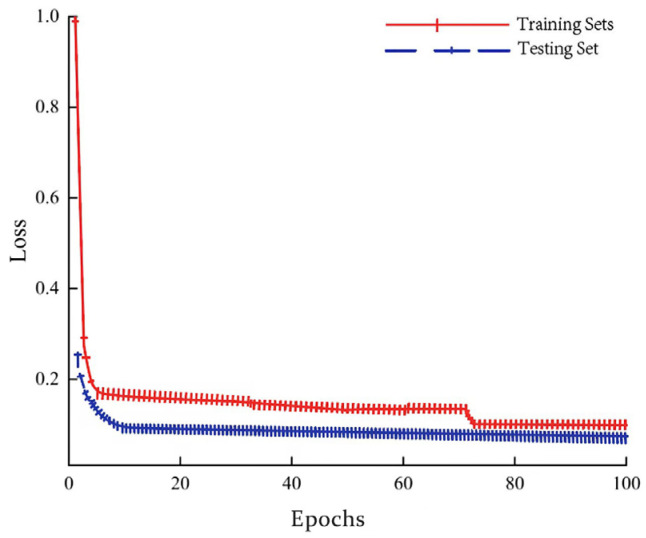
Loss value change curve during training.

The laboratory self-made coal and gangue dataset and the underground working face coal and gangue dataset are labeled using Labelimg image-annotation software. In order to ensure the rigor of the experiment, the Labelimg image-annotation software is used to complete the annotation of the sample image by manual annotation. The dataset is divided according to (training set + verification set): test set = 9:1, and the training set is set to: verification set = 9:1. The size of the training input image is 640 × 640, and the two-stage fine-tuning training strategy is adopted. Firstly, the training batch size is set to 30, the epochs are set to 50, the initial learning rate is 0.01, the minimum learning rate is 0.0005, the weight attenuation is 0.001, and the sgd optimizer and cosine descent learning rate are used. Then, the training batch size is set to 10, the epochs are set to 50, and the optimizer, learning rate, and weight attenuation coefficient remain unchanged. The loss value l of the model training process is shown in Figure 5, and the model reaches a convergence state at the 73rd generation.

**Figure 6:**
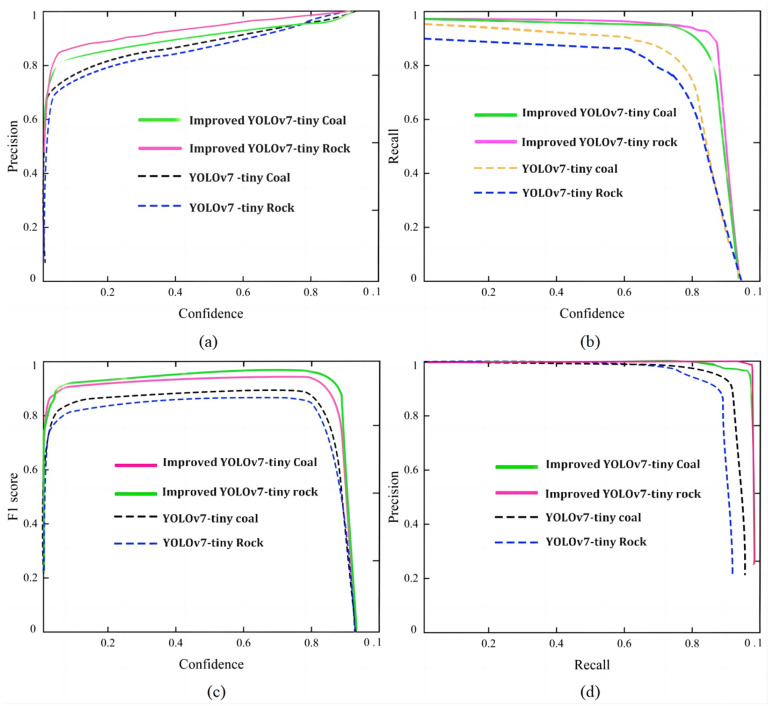
Improved YOLOv7-tiny and YOLOv7-tiny model coal and gangue identification evaluation index change curves. (**a**) Precision curve; (**b**) Recall rate curve; (**c**) F1 value curve; (**d**) Precision and recall rate curves.

The results of the coal–gangue recognition of the improved YOLOv7-tiny model and the YOLOv7-tiny model are shown in Figure 6; the data are shown in Table 1. Figure 6 describes the changes in the precision, recall rate, F1 value, and average accuracy of the improved YOLOv7-tiny model and the YOLOv7-tiny model in identifying coal–gangue under different confidence levels. It can be seen that the precision of the improved YOLOv7-tiny model in identifying coal–gangue is much better than that of the YOLOv7-tiny model. Under the condition of a given confidence = 0.5, the precision, recall rate, and F1 value of the improved YOLOv7-tiny model for identifying coal–gangue are 4.33%, 8.14%, and 0.065 higher than those of the YOLOv7-tiny model, respectively, and the average accuracy is increased by 7.53%. This shows that the improved YOLOv7-tiny model has a stronger recognition ability on the coal and gangue dataset, and the average accuracy can reach 97.54%.

### 3.3. Ablation Experiment

In order to further verify the effectiveness of each module, ablation experiments were performed on each module of the improved network. The use of each module and the experimental results are shown in Table 2. The average accuracy of the model (mAP@0.5), the number of parameters (Params), and the processor rate (GFLOPs) are used as the main evaluation indicators [25,26]. It can be seen from the table that the mAP@0.5 of the original YOLOv7-tiny network on the dataset of this paper is 92.0%, the model parameter is 6.03 M, and the calculation amount is 13.28 G. After adding the coordinate attention mechanism to the two feature maps in the middle of the backbone network, the mAP@0.5 increased by 0.4%, indicating that the coordinate attention mechanism can better extract coordinate and channel information and improve the feature expression ability of the model. After adding the contextual transformer module after the pooling pyramid, the mAP@0.5 increased by 0.9%, indicating that the contextual transformer module obtained certain global information and improved the ability to capture defect features. When using the Bi-ELAN, the mAP@0.5 increased by 0.6%, indicating that the improved feature fusion method was helpful in improving the network performance. When the Focal-EIOU was used, the mAP@0.5 is increased by 0.7%, indicating that the Focal-EIOU has a good effect on reducing the loss oscillation caused by low-quality samples and balancing difficult and easy samples. Experiments 6 and 7 further verified the effectiveness of the contextual transformer and coordinate attention mechanism. After removing these two modules, the mAP@0.5 was reduced by 0.3% and 0.1%, respectively. The parameter quantity of the improved model is 6.62 M, which is 0.59 M higher than that of the original algorithm. The calculation amount is 13.78 G, which is 0.5 G higher than that of the original algorithm. The mAP@0.5 increased from 92.0% to 93.2%. In general, this experiment improves the overall mAP@0.5 value while increasing a small amount of parameter quantity and calculation amounts.

## 4. Comparative Experiments of Different Models to Identify Coal and Gangue Images

**Figure 7:**
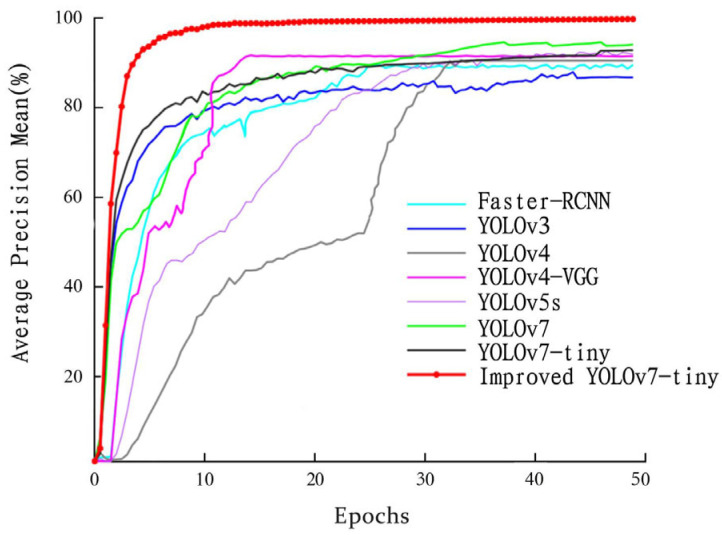
The mAP curves for different model comparison experiments.

In order to objectively evaluate the superiority of the improved YOLOv7-tiny model proposed in this paper in the recognition performance of coal and gangue, a comparative experiment with other classical models (Faster-RCNN, YOLOv3, YOLOv4, YOLOv4-VGG, YOLOv5s, and YOLOv7) was designed for the analysis. All models are carried out in an experimental environment with the same parameter configurations. All models are trained using the same dataset; that is, the three datasets of the public dataset, laboratory self-made coal and gangue dataset, and the coal and gangue dataset of the underground working face of a coal mine. At the same time, the ratio of datasets used by all models is the same, which is carried out according to the ratio of (training set + verification set): test set = 9:1, and the training set is set to: verification set = 9:1. In this paper, the Params, the number of floating-point operations (FLOPs), the FPS, and the average accuracy mean are used as evaluation indices to evaluate the performance of different models. The experimental results are shown in Table 3, and the average accuracy mean curve of different model comparison experiments is drawn, as shown in Figure 7.

Compared with the YOLOv3, YOLOv4, YOLOv4-VGG, YOLOv5s, and YOLOv7, the parameters of the improved YOLOv7-tiny model are greatly reduced, and slightly higher than the YOLOv7-tiny and YOLOv4-VGG. The number of floating-point operations is only higher than the YOLOv4, and far lower than the Faster-RCNN, YOLOv4-VGG, YOLOv5s, and YOLOv7. The number of frames per second of the image processed by the model is also in the forefront. It is significantly higher than the YOLOv5s and YOLOv7, and slightly lower than the YOLOv3 and YOLOv4-VGG. Compared with the other seven models, the improved YOLOv7-tiny model achieved the highest recognition accuracy of 97.54%. The results of the comprehensive comparative experiments show that the improved YOLOv7-tiny model proposed in this paper achieves a good balance between the accuracy and speed of coal and gangue recognition, and can realize the real-time and accurate recognition of coal and gangue.

**Figure 8:**
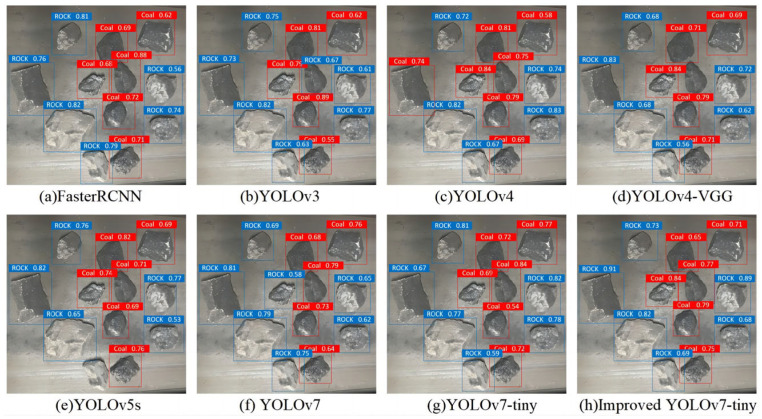
Coal and gangue identification results from the different models.

The recognition results of different models on coal and gangue test sets are shown in Figure 8. From the recognition results, the improved YOLOv7-tiny model can accurately classify and identify coal and gangue, while the other models have different degrees of missed detection. From the perspective of recognition accuracy, the improved YOLOv7-tiny model has the highest confidence level in identifying coal and gangue among all the comparison models. The number in the image detection box represents the confidence of the model recognition, and the higher the confidence, the more accurate the model prediction. It is found, in Figure 8, that the YOLOv3 model mistakenly identifies a piece of coal as rock, the YOLOv4-VGG omits the identification of a piece of coal, the YOLOv5s model omits the identification of a piece of gangue, and the YOLOv7 model mistakenly identifies a piece of coal as gangue. Through the comprehensive comparison, it is found that the improved YOLOv7-tiny model has the best recognition effect.

## 5. Field Application Effect in an Underground Coal Mine

**Figure 9:**
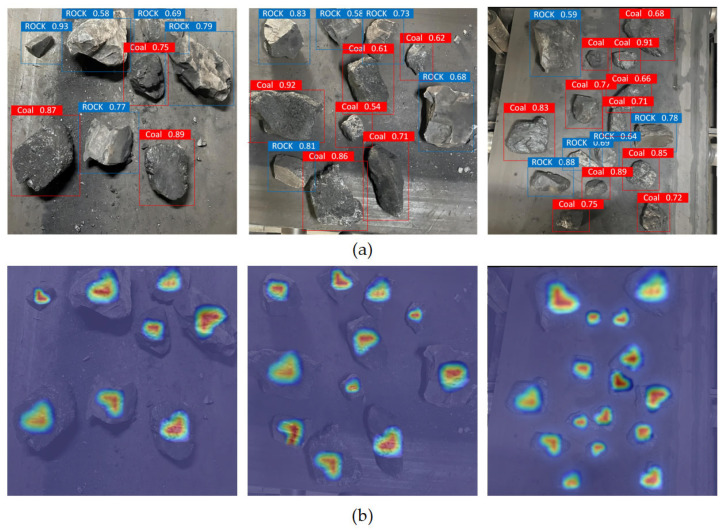
Image recognition results for coal and gangue on belt conveyor at the working face. (**a**) Identification results of coal and gangue; (**b**) Location rendering of the coal and gangue.

In order to verify the feasibility of the improved YOLOv7-tiny model proposed in this paper in the practical application of the coal mine underground working face, more than 1000 coal and gangue images were collected from the belt conveyor of the 5013 fully mechanized mining face in the Xiaoyu Coal Mine, Shanxi Province, and expanded by the data enhancement method. In order to expand the scale of the dataset and enhance the characteristics of the dataset, the data enhancement method was used to expand the dataset of the coal and gangue in the underground working face of the coal mine. The data enhancement methods used in this paper include image size scaling, rotation, pixel translation, changing the contrast, perspective transformation, and adding noise. Finally, a dataset containing 6397 coal and gangue images was formed, and the training set and test set were divided according to the ratio of 9:1. Three images were randomly selected from the recognition results of the experimental test set as the experimental results, as shown in Figure 9. The red rectangular frame is marked with coal blocks, and the blue rectangular frame is marked with gangue. The position of the coal and gangue is rendered by the heat map. It can be seen that the improved YOLOv7-tiny model has a good global perception ability, which can accurately identify the characteristics of the coal and gangue and mark the position and confidence level.

## 6. Conclusions

(1) This paper proposes a coal and gangue recognition model based on the improved YOLOv7-tiny target detection algorithm. The average recognition accuracy of the model on the coal and gangue dataset can reach 97.54%, which is 7.47% higher than that of the YOLOv7-tiny. The parameter quantity of the model is 30.75 M, and the FPS is 24.73 f·s^−1^. This model can quickly and accurately identify coal and gangue.

(2) This paper proposes three model improvement methods. The coordinate attention mechanism is introduced to improve the feature expression ability of the model. The contextual transformer module is added after the spatial pyramid pooling structure to improve the feature extraction ability of the model while ensuring a small increase in the number of parameters. Based on the idea of the weighted bidirectional feature pyramid, the four branch modules in the ELAN module are weighted and cascaded to improve the recognition ability of the model for useful features. Compared with the YOLOv7-tiny model, the number of parameters and the number of floating-point operations of the improved YOLOv7-tiny model are reduced by 17.23% and 60.41%, and the recognition accuracy of coal and gangue is improved by 7.46%, which achieves a good balance between the recognition accuracy and the speed of the model.

(3) The improved YOLOv7-tiny model proposed in this paper has achieved good results in the accuracy and speed of coal and gangue recognition. It can achieve 24 recognitions per second and maintain a recognition rate of more than 97%, which can achieve the real-time accurate recognition of coal–gangue. At the same time, the coal mine underground test results show that the improved YOLOv7-tiny model can well-identify the coal and gangue on the belt conveyor in the fully mechanized mining area of the coal mine, verify the feasibility of the model application, and provide effective technical means for the accurate identification of coal and gangue.

## Figures and Tables

**Table 1:** Comparison of the improved YOLOv7-tiny and the YOLOv7-tiny algorithm coal and gangue identification results.

Identification Category	Improved YOLOv7-Tiny (Confidence = 0.5)				YOLOv7-Tiny (Confidence = 0.5)			
	Precision	Recall Rate	F1 Value	Average Accuracy	Precision	Recall Rate	F1 Value	Average Accuracy
Coal	90.56%	96.75%	0.95	96.63%	88.72%	90.07%	0.9	92.45%
Gangue	93.98%	98.31%	0.94	98.39%	87.15%	88.71%	0.86	87.56%
Mean Value	92.27%	97.53%	0.945	97.54%	87.94%	89.39%	0.88	90.01%

**Table 2:** Results of the ablation experiment.

Experiment	CA	CoT	Bi-ELAN	Focal-EIOU	mAP@0.5/%	Params/M	GFLOPs/G
1	-	-	-	-	92.0	6.03	13.28
2	√	-	-	-	92.4	6.04	13.32
3	-	√	-	-	92.9	6.61	13.74
4	-	-	√	-	92.6	6.03	13.28
5	-	-	-	√	92.7	6.03	13.28
6	√	-	√	√	92.9	6.04	13.32
7	-	√	√	√	93.1	6.61	13.74
8	√	√	√	√	93.2	6.62	13.78

**Table 3:** Performance comparison of different coal and gangue identification models.

Model	Params (M)	FLOPs (×10^9^)	FPS (f·s^−1^)	Average Precision Mean
Faster-RCNN	60.34	66.50	25.91	87.60
YOLOv3	28.63	119.41	23.62	85.91
YOLOv4	39.16	38.89	20.37	88.96
YOLOv4-VGG	23.69	112.73	25.67	89.37
YOLOv5s	65.19	60.55	21.33	90.12
YOLOv7	46.07	114.84	14.28	92.31
YOLOv7-tiny	37.33	106.97	14.59	90.07
Improved YOLOv7-tiny	30.75	41.25	24.73	97.54

## Data Availability

The experimental data used to support the findings of this study are included within the article.
